# Evaluation of FMR4, FMR5 and FMR6 Expression Levels as Non-Invasive Biomarkers for the Diagnosis of Fragile X-Associated Primary Ovarian Insufficiency (FXPOI)

**DOI:** 10.3390/jcm11082186

**Published:** 2022-04-14

**Authors:** Maria Isabel Alvarez-Mora, Ines Agusti, Robin Wijngaard, Estefania Martinez-Barrios, Tamara Barcos, Aina Borras, Sara Peralta, Marta Guimera, Ana Goday, Dolors Manau, Laia Rodriguez-Revenga

**Affiliations:** 1 Biochemistry and Molecular Genetics Department, Hospital Clinic of Barcelona and Institut d’Investigacions Biomèdiques August Pi i Sunyer (IDIBAPS), 08036 Barcelona, Spain; mialvarez@clinic.cat (M.I.A.-M.); wijngaard@clinic.cat (R.W.); estefaniamartinezbarrios@gmail.com (E.M.-B.); tamara_barcos@hotmail.com (T.B.); 2 CIBER of Rare Diseases, Instituto de Salud Carlos III, 28029 Madrid, Spain; 3 Clinical Institute of Gynecology, Obstetrics and Neonatology (ICGON), Hospital Clinic of Barcelona and Institut de Investigacions Biomèdiques August Pi iSunyer (IDIBAPS), 08036 Barcelona, Spain; iagustis@clinic.cat (I.A.); aborras1@clinic.cat (A.B.); speralta@clinic.cat (S.P.); mguimera@clinic.cat (M.G.); goday@clinic.cat (A.G.); dmanau@clinic.cat (D.M.)

**Keywords:** FMR4, FMR5, FMR6, *FMR1* gen, CGG repeat, *FMR1* premutation and FXPOI

## Abstract

Female *FMR1* (Fragile X mental retardation 1) premutation carriers are at risk for developing fragile X-associated primary ovarian insufficiency (FXPOI), a condition characterized by amenorrhea before age 40 years. Not all women with a *FMR1* premutation suffer from primary ovarian insufficiency and nowadays there are no molecular or other biomarkers that can help predict the occurrence of FXPOI. Long non-coding RNAs (lncRNAs) comprise a group of regulatory transcripts which have versatile molecular functions, making them important regulators in all aspects of gene expression. In recent medical studies, lncRNAs have been described as potential diagnostic biomarkers in many diseases. The present study was designed to determine the expression profile of three lncRNAs derived from the *FMR1* locus, FMR4, FMR5 and FMR6, in female *FMR1* premutation carriers in order: (i) to determine a possible role in the pathogenesis of FXPOI and (ii) to investigate whether they could serve as a biomarker for the diagnosis of FXPOI. FMR4, FMR5 and FMR6 transcripts levels were evaluated in total RNA extracted from peripheral blood by digital droplet PCR and compared between *FMR1* premutation carriers with FXPOI and without FXPOI. The diagnostic value of lncRNAs was evaluated by receiver operating characteristic (ROC) analysis. Results revealed a significant association between FXPOI and high expression levels of FMR4. No association was obtained for FMR5 or FMR6. ROC curve analysis revealed that FMR4 can distinguish *FMR1* premutation carrier with FXPOI with a diagnostic power of 0.67. These findings suggest a potential role of FMR4 as a possible biomarker for FXPOI.

## 1. Introduction

The *FMR1* (Fragile X mental retardation 1) gene (OMIM*309550) contains a CGG trinucleotide repeat in the 5′ untranslated region [1,2,3]. Among individuals in the general population, the number of CGG repeats is polymorphic and varies between 6 and 44. In this situation the *FMR1* gene is active and there is synthesis of the Fragile Mental Retardation Protein (FMRP). Repeats in this size interval are stable when transmitted from generation to generation. In contrast, when the number of CGGs exceeds 200 repeats (full mutation) the *FMR1* gene is silenced resulting in the absence of FMRP and the clinical manifestations of fragile X syndrome (FXS, OMIM#300624; ORPHA:908): intellectual disability and characteristic dysmorphic features [4]. Finally, individuals with alleles between 55 and 200 CGG repeats are the so-called *FMR1* premutation carriers. In this situation, there is increased transcription of the *FMR1* mRNA transcript, and slightly reduced synthesis of FMRP [5]. These repeat alleles are unstable and tend to increase in each cell division, conferring an increased risk of having FXS affected offspring. 

*FMR1* premutation occurs in the general population with an estimated frequency that range from 1 in 800 to 1200 males and from 1 in 250 to 400 females [6]. These individuals are at risk for fragile X-associated premature ovarian insufficiency (FXPOI) in females [7], and fragile X-associated tremor/ataxia syndrome (FXTAS) in both, males and females [8].

FXPOI symptoms include the cessation of ovarian function before the age of 40 years, ovarian dysfunction, and decreased fertility as evidenced by abnormal ovarian reserve biomarkers and reduced ovarian response to controlled ovarian hyperstimulation [9,10,11]. FXPOI occurs in ~20% of women with the *FMR1* premutation, compared to only 1% of the general population [12]. Women with the *FMR1* premutation not only struggle with the possibility of developing FXPOI but also with the risk of passing on the full mutation to their offspring. 

Long non-coding RNAs (lncRNAs), defined as untranslated RNA molecules greater than 200 nucleotides in length, can be derived from sense or antisense strands within protein-coding genes, intergenic regions, or pseudogenes [13,14,15]. A lot of evidence has been accumulated showing that lncRNAs have versatile molecular functions, making them important regulators in all aspects of gene expression (reviewed in [16]). The biological mechanisms of lncRNAs are multiple. They can interact with DNA, RNA and protein as well as with other biological molecules, regulating the gene expression trough controlling processes such as protein synthesis, RNA maturation and transport, or the chromatin structure [17]. Moreover, lncRNAs participate in diverse biological processes, such as development, differentiation, energy metabolism, apoptosis and angiogenesis; controlling every level of gene expression pathway [18]. These particularities make them good candidate biomarkers for several diseases.

Several lncRNAs are derived from the *FMR1* gene locus: FMR4, FMR6 and the ASFMR1 in the antisense direction and FMR5 in the sense direction (reviewed in [19]). Previous studies have described different expression levels of these lncRNAs among *FMR1* permutation carriers, suggesting a functional association with fragile X-associated disorders [20,21,22,23,24]. Although the *FMR1* premutation is the major risk factor for developing FXPOI, there are still some unknown genetic, epigenetic or environmental factors that might be affecting gene penetrance. On the basis of this observation, we aim to determine the expression profile of FMR4, FMR5 and FMR6 in female *FMR1* premutation carriers in order to determine a possible role in the pathogenesis of FXPOI. 

## 2. Material and Methods

### 2.1. Subjects

For this study, 36 female *FMR1* premutation carriers registered in the FXS database from the Department of Biochemistry and Molecular Genetics, Hospital Clinic of Barcelona were selected. The cases met the following criteria: 20 women diagnosed with FXPOI (development of amenorrhea due to disruption of ovarian function before the age of 40 years) carrying the *FMR1* premutation (CGG repeats between 55–200) and 16 non-FXPOI *FMR1* premutation carriers with a reported normal ovarian function (regular cycles between 24–35 days) over the age of 40 years. This study was approved by the Institutional Ethical Review Board of Hospital Clinic, Barcelona. All patients that were included in this study signed a written informed consent.

### 2.2. RNA Extraction and cDNA Synthesis

Total RNA isolation was performed from blood using the PAXgene^®^ Blood RNA Kit (Qiagen, Hilden, Germany) according to the manufacturer’s protocols. A Qubit RNA IQ assay (ThermoFisher Scientific, Waltham, MA, USA) was used to determine the RNA concentration. Its integrity was verified with the Bioanalyzer 2100 (Agilent, Santa Clara, CA, USA). The cDNA was synthesized from 350 ng RNA using the High-Capacity cDNA reverse transcription Kit (Applied Biosystems, Waltham, MA, USA), following the manufacturer’s instructions. 

### 2.3. Digital Droplet Polymerase Chain Reaction (ddPCR)

LncRNA expression analysis was performed by ddPCR technology, which is an improvement of conventional PCR. Using a droplet generator, the sample is diluted and divided into multiple aliquots and subjected to endpoint PCR. Amplification occurs in each individual partition, which contains an individual nucleic acid molecule. A fluorescence signal is produced in each droplet with the target molecule. Quantification of cDNA molecules relies on the ability of the ddPCR system to determine the number of target molecules by the Poisson statistical analysis of “positive” (containing amplified target) and “negative” (no amplified target detected) droplets [25]. The assay was performed using the QX200™ ddPCR™ EvaGreen Supermix (Bio-Rad, Hercules, CA, USA), 100 nM specific primers targeted to FMR4 (f:5′-ACCAAACCAAACCAAACCAA-3′ and r:5′-GTGGGAAATCAAATGCATCC-3′), FMR5 (f:5′-AATGCTGGCAGTCGTTTCTT-3′ and r:5′-TTGACGGAGCATCTATCGTG-3′), FMR6 (f:5′-AGCACTTCAGGGCAGATTTT-3′ and r:5′-TGGTGAATGATCACCCAATG-3′) and the housekeeping gene GAPDH (f:5′-TCTCCTCTGACTTCAACAGCGAC-3′ and r:5′- CCCTGTTGCTGTAGCCAAATTC-3′) and 9 μL of the diluted cDNA sample. Droplet emulsion formation was performed by mixing 20 μL of reaction with 70 μL of droplet generation oil using a microfluidic droplet generation cartridge and QX200 Droplet Generator (Bio-Rad). End-point PCR amplification was performed using a C1000 Thermal Cycler (Bio-Rad). Primers for FMR5 and GAPDH were designed with Primer-3 (http://bioinfo.ut.ee/primer3-0.4.0/) (accessed on 10 May 2019). Primer sequences for FMR4 and FMR6 were extracted from Elizur and co-workers [24]. In order to normalize lncRNAs copies relative to nuclear DNA, the *GAPDH* gene was used as reference gene.

Results were analyzed with QuantasoftTM Software (Bio-Rad). Target RNA concentrations were calculated using the Poisson statistics and the expression of the lncRNA was reported as [copies/cell] corrected for the expression of the reference gene *GAPDH*. Expression levels are shown as transcripts per ten thousand cells.

### 2.4. FMR1 Molecular Parameters

CGG repeat analysis and X-chromosome inactivation (XCI) pattern were determined using the AmplideX^®^ PCR/CE *FMR1* and AmplideX^®^ mPCR *FMR1* kits, following manufacturer’s recommendations (Asuragen, Austin, TX, USA). Skewed XCI status was considered with a threshold value greater than 90% [26]. *FMR1* mRNA quantification was performed as previously described [27]. 

### 2.5. Statistical Analysis

Statistical analysis was carried out using the IBM^®^ SPSS^®^ Statistics software version 25 (SPSS, Chicago, IL, USA) and the open-source computing environment R version 4.0.2 (R Foundation for Statistical Computing, Vienna, Austria). The pROC, ggplot2, MASS and caret R packages were used for assessment of the expression data. Results were expressed as mean ± standard deviation (SD). Statistical significance of differences between means or medians was examined using the parametric *t*-test or the non-parametric Mann–Whitney U test. The discriminatory ability of the lncRNAs to separate FXPOI from non-FXPOI women was assessed using receiver operating characteristic (ROC) curves and by calculating the area under the curve (AUC). Cut-off values were decided based on the optimal combined sensitivity and specificity threshold (Youden Index). The Odds Ratio (OR) was calculated to assess the association between the increased biomarker and FXPOI. Additionally, a multivariate logistic regression model was generated including FMR4 expression levels corrected by CGG repeats. Significance was accepted for *p*-value < 0.05.

## 3. Results

### 3.1. Expression Levels of FM4, FMR5 and FMR6 in FMR1 Premutation Carriers

Thirty-six *FMR1* premutation female carriers were recruited; 20 with FXPOI and 16 without FXPOI. There were no differences between the two groups regarding patients’ CGG repeat expansion or *FMR1* mRNA expression levels (Table 1). As for the patients’ age at time of analysis, statistically significant differences were obtained (*p* = 0.03). This difference can be attributed to a bias in the recruitment of women without FXPOI. Older women were included in this cohort in order to make sure that they did not have ovarian dysfunction. X-chromosome inactivation (XCI) pattern was also examined and compared between the FXPOI and non-FXPOI groups. Results evidenced that both groups showed a similar random XCI pattern (data not shown).

**Figure 1:**
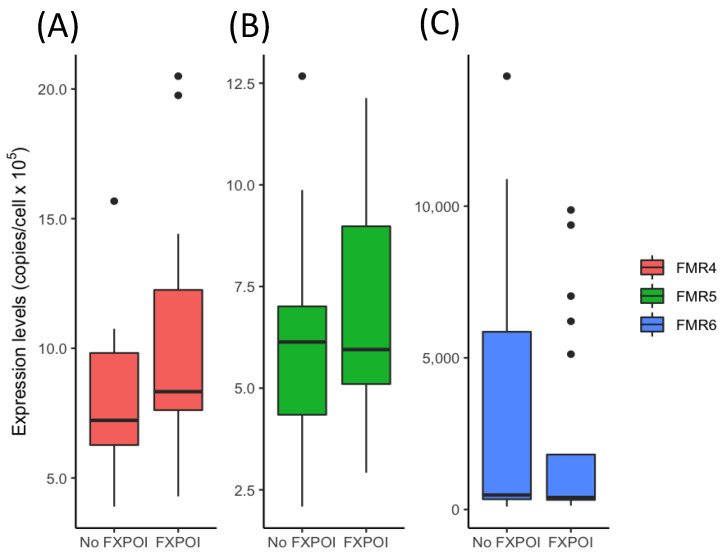
Expression levels of FMR4, FMR5 and FMR6 were compared between *FMR1* premutation carriers with FXPOI and without FXPOI. (**A**) The expression level of FMR4, (**B**) The expression level of FMR5 and (**C**) The expression level of FMR6. None of the comparisons were statistically significant (*p* > 0.05).

FMR4, FMR5 and FMR6 transcripts levels were evaluated in total RNA extracted from peripheral blood by ddPCR and compared between *FMR1* premutation carriers with FXPOI and without FXPOI. Results showed no significant differences between groups for none of the lncRNAs analyzed (*p* = 0.09 for FMR4; *p* = 0.46 for FMR5 and *p* = 0.56 for FMR6) (Figure 1 and Table S1). Subsequently, and since the expression of the three lncRNAs varies over a wide range, we compared *FMR1* premutation carriers with and without FXPOI among stratified subgroups based on expression levels. These subgroups consisted of low, medium and high expression levels for FMR4, FMR5 and FMR6. When comparing the distribution of lncRNAs expression levels (low, medium and high) between *FMR1* premutation carrier groups (with and without FXPOI), significant results were obtained for FMR4 (*p* = 0.039). As shown in Table 2, while 30% (6/20) of FXPOI women showed high levels of FMR4 expression, only 10% (2/16) of women without FXPOI were found to have similar levels. Therefore, evidence was obtained for an association between FMR4 transcript levels and the development of FXPOI. A similar association was also found for FMR5, although not statistically significant. Contrary, for FMR6, no significant evidence of an association was obtained (Table 2).

**Figure 2:**
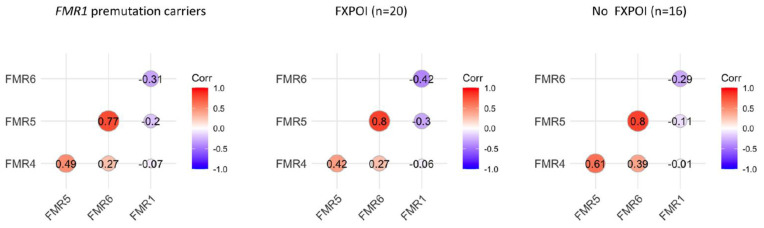
Correlation between expression levels of FMR4, FMR5 and FMR6 was evaluated among total *FMR1* premutation carriers as well as groups of women with FXPOI and without FXPOI.

In addition, the correlation between the expression levels of FMR4, FMR5, FMR6 and the *FMR1* gene was appraised among the total number of female *FMR1* premutation carriers as well as in the distinct groups with and without FXPOI (Figure 2). A high correlation was found between FMR5 and FMR6 expression levels while the correlation of these two lncRNAs with FMR4 was lower. All lncRNAs correlated negatively with respect to *FMR1* expression. The correlation pattern did not change when FXPOI and non-FXPOI females were considered separately, suggesting that their relative expression behavior is not substantially affected by the presence of FXPOI.

### 3.2. Diagnostic Value of FMR4 for FXPOI

**Figure 3:**
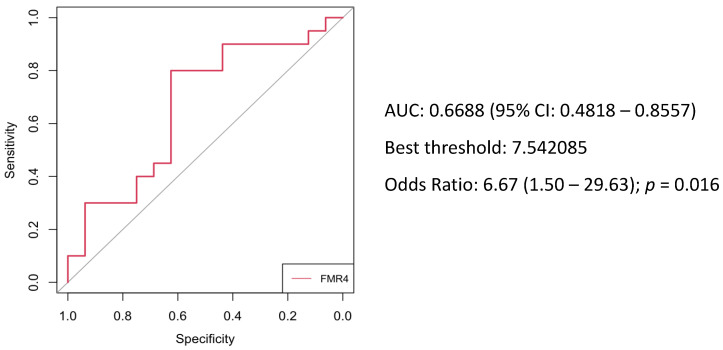
ROC curve for assessment of diagnostic power of FMR4 among total *FMR1* premutation female carriers. The area under the curve (AUC) was 0.67.

Since significant differences were obtained in the distribution of FMR4 expression levels between women with FXPOI and without FXPOI, the diagnostic value of FMR4 was assessed by establishing a ROC curve. As can be observed in Figure 3, the curve had an AUC value of 0.67 (95% CI 0.45–0.86). A sensitivity of 0.80 and specificity of 0.63 were achieved at the optimal threshold. These data indicate that FMR4 expression levels had a certain diagnostic value for FXPOI. FMR4 levels above the established threshold conferred a significantly increased risk of developing FXPOI (OR: 6.67 95% CI: 1.5–29.63, *p* = 0.016).

### 3.3. Association of lncRNAs Expression Levels with FMR1 CGG Repeat Size

**Figure 4:**
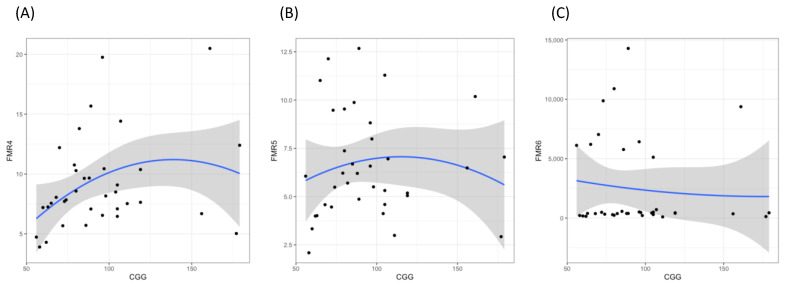
lncRNAs expression level in peripheral blood of *FMR1* premutation carriers according to the number of CGG repeats. (**A**) FMR4 expression levels, (**B**) FMR5 expression levels, and (**C**) FMR6 expression levels.

We evaluated the correlation between the expression levels of the lncRNAs and the CGG repeat size (Figure 4). Expression of FMR4 and FMR5 showed a non-linear distribution, although the association was only statistically significant for FMR4 (*p* = 0.037). The highest levels of FMR4 were detected in women with 80–99 CGG repeats (Figure 4A). For FMR6, as the expression levels showed a wide distribution range, no evidence of an association was inferred (*p* = 0.9) (Figure 4C).

A multivariate logistic regression analysis was performed to explore whether the correction of FMR4 expression levels for the number of CGG repeats (as a curvilinear confounder) could improve the prediction of FXPOI. Although, the inclusion of both parameters also showed a similar 0.67 AUC, FMR4 expression did not show a significant adjusted odds ratio in the model (*p* = 0.15).

## 4. Discussion

Little is known about the disease pathology underlying FXPOI. Although the *FMR1* premutation is the major risk factor, there are still some unknown genetic, epigenetic or environmental factors that might be affecting gene penetrance. Current research aims to identify molecular or other biomarkers that can predict the occurrence of FXPOI and help women with the *FMR1* premutation to make decisions regarding their reproductive and family planning. LncRNAs have attracted a lot of attention in recent medical studies since emerging evidence suggests that they could be used as diagnostic biomarkers in many diseases such as cancer [28], cardiovascular or neurodegenerative disorders [29,30]. FMR4, FMR5 and FMR6 comprise a group of *FMR1*-derived lncRNAs which might partake to aspects of the clinical presentation of the Fragile X-associated disorders [21,22,23,24]. In fact, it has been described that the expression of these lncRNAs is different in both FXS patients and *FMR1* premutation carriers. In brain tissue from FXS patients both, FMR4 and FMR6 expression have been found to be down-regulated [21,22]. On the other hand, in *FMR1* premutation carriers whereas the FMR4 expression has been described up-regulated [22], the FMR6 expression has been found down-regulated [21].

On the basis of these observations, this study investigated FMR4, FMR5 and FMR6 expression levels in peripheral blood of female *FMR1* premutation carriers with and without FXPOI in order to explore their feasibility as potential biomarkers of FXPOI. For this purpose, the ddPCR technique was used, as it is a highly sensitive and specific method for absolute quantification of transcript gene expression per cell.

The results showed a wide range of expression levels for all three lncRNAs analyzed, leading to a non-significant difference when comparing mean expression levels between female *FMR1* premutation carriers with FXPOI and without FXPOI. We further assessed pairwise correlation between the expression of FMR4, FMR5, FMR6 and *FMR1* mRNA transcripts. Again, no differences were observed when comparing women with FXPOI and without FXPOI; suggesting that pairwise correlations between expressions of these lncRNAs and the *FMR1* gene might not be affected by the presence of FXPOI (Figure 2). However, a statistically significant difference was obtained for FMR4 (*p* = 0.039) when comparing both groups stratified by expression levels (low, medium or high), indicating a disequilibrium in the distribution. Among all the *FMR1* premutation carriers presenting higher FMR4 expression levels, 86% (6/7) developed FXPOI whereas only 29% (1/6) did not. ROC curve analysis revealed that FMR4 can modestly distinguish female *FMR1* premutation carriers with and without FXPOI with an AUC of 0.67. Although caution must be taken due to the limited sample size, our results showed an association between high FMR4 expression levels and FXPOI (OR 6.67, 95% CI = 1.5–29.63, *p* = 0.016), suggesting that this lncRNA could be a possible marker for FXPOI among female *FMR1* premutation carriers.

To date, only CGG repeat size has been associated with the risk of developing FXPOI in a non-linear way [31,32,33]; leading to the highest risk for FXPOI in the mid-range of the repeat expansion (~80–99 CGG). Interestingly, our findings, although not statistically significant, also showed a non-linear correlation between the FMR4 and FMR5 expression levels and the *FMR1* repeat size; yielding higher levels of both, FMR4 and FMR5 expression, in the mid-range. This correlation was not detected for FMR6 (Figure 4C). Nevertheless, since FMR6 showed the highest range of expression levels, it cannot be completely discarded. To our knowledge, only Elizur and co-workers [24] have previously analyzed the transcript levels of FMR4 and FMR6 in granulosa cells of *FMR1* premutation carriers in order to determine a putative role in the pathogenesis of FXPOI. Contrary to our results, the authors did not observe any association neither between granulosa cells FMR4 expression levels nor between the number of CGG repeats in the *FMR1* gene. On the other hand, whereas the FMR6 expression levels were not significantly different between *FMR1* permutation carriers and controls, they reported a significant non-linear association between the number of CGG repeats and FMR6 levels in the granulosa cells [24]. Although these results are not in line with ours, it has to be taken into consideration that the methodology and tissue used to measure the lncRNAs levels were different. Whereas Elizur et al. [24] used qRT-PCR and granulosa cells, we used peripheral blood and ddPCR. Moreover, although the sample size was similar in both studies, they compared *FMR1* premutation carriers against controls and we compared *FMR1* premutation carriers with FXPOI against those without FXPOI. Finally, another important difference is that their cohort lack of *FMR1* premutation carriers with *FMR1* repeat size above 150 CGGs, which could somehow bias the results.

Our study, although exploratory, has two main limitations. First, the sample size, which is not large enough to provide reliable evidence for an association between FMR4, FMR5 and FMR6 expression levels and FXPOI. However, our study provides statistically significant results, highlighting a potential role of FMR4 in predicting FXPOI. Second, the age differences between groups and the fact that *FMR1* premutation women with FXPOI had already developed ovarian dysfunction. Thus, it would be necessary to replicate our findings in other female *FMR1* premutation cohorts and, ideally, in a longitudinal study, in order to make sure that age is not affecting lncRNAs expression levels. If validated in other populations, these results might provide evidence of a potential role of FMR4 as a possible biomarker for FXPOI.

## Figures and Tables

**Table 1:** Clinical and molecular characteristics of individuals recruited in the study.

	*FMR1* Premutation with FXPOI (n = 20)	*FMR1* Premutation without FXPOI (n = 16)	*p*-Value
Age (mean ± SD, years)	41 ± 6.9	47 ± 8.3	0.03 *
CGG repeat (mean ± SD)	100 ± 35	88 ± 26	0.3
*FMR1* mRNA (mean ± SD)	1.5 ± 0.8	1.8 ± 1.3	0.3

Significance: * *p* < 0.05. The exact *p*-values were calculated with the U-Mann Whitney test.

**Table 2:** Distribution of *FMR1* premutation carriers with and without FXPOI based on FMR4, FMR5 and FMR6 expression levels.

	**FMR4 expression level**			***p*-Value**
	**1–7**	**7–12**	**>12**	0.039 *
FXPOI (n = 20)	2 (10%)	12 (60%)	6 (30%)	
No FXPOI (n = 16)	7(44%)	8 (50%)	1 (6%)	
	**FMR5 expression level**			0.14
	**1–5**	**5–10**	**>10**	
FXPOI (n = 20)	5 (25%)	10 (50%)	5 (25%)	
No FXPOI (n = 16)	7 (44%)	8 (50%)	1 (6%)	
	**FMR6 expression level**			0.556
	**<400**	**400–1000**	**>1000**	
FXPOI (n = 20)	10 (50%)	5 (25%)	5 (25%)	
No FXPOI (n = 16)	6 (38%)	5 (31%)	5 (31%)	

Significance: * *p* < 0.05. The exact *p*-values were calculated with the Fisher exact test.

## Data Availability

The analyzed data sets generated during the study are available from the corresponding author on reasonable request.

## Supplementary Materials

The following supporting information can be downloaded at: https://www.mdpi.com/article/10.3390/jcm11082186/s1, Table S1: Expression levels of FMR4, FMR5 and FMR6 measured in peripheral blood of *FMR1* premutation carriers with and without FXPOI.
