# Supplementary material for: Evaluation of FMR4, FMR5 and FMR6 Expression Levels as Non-Invasive Biomarkers for the Diagnosis of Fragile X-Associated Primary Ovarian Insufficiency (FXPOI)
Source: J Clin Med. 2022 Apr 14;11(8):2186. doi: 10.3390/jcm11082186 (PMC9025681; doi:10.3390/jcm11082186)
Supplement: Supplementary file 1 [file jcm-11-02186-s001.zip › jcm-1622783-supplementary.pdf]

Supplementary Table S1. Expression levels of FMR4, FMR5 and FMR6 measured in peripheral blood of *FMR1* premutation carriers with and without FXPOI.

	**FXPOI (n=20)**	**Non-FXPOI (n=16)**	***P* value**
FMR4	8.33 (7.62 – 12.25)	7.22 (6.27 – 9.82)	0.09
FMR5	5.95 (5.10 – 8.98)	6.14 (4.35 – 7.01)	0.46
FMR6	397.00 (318.65 – 1813.08)	477.6 (339.66 – 5859.01)	0.56

The exact p-values were calculated with the Mann-Whitney U test.
